# Vascular maturity of type 1 and type 2 choroidal neovascularization evaluated by optical coherence tomography angiography

**DOI:** 10.1371/journal.pone.0216304

**Published:** 2019-04-29

**Authors:** Yuyako Nakano, Keiko Kataoka, Jun Takeuchi, Ai Fujita, Hiroki Kaneko, Hideyuki Shimizu, Yasuki Ito, Hiroko Terasaki

**Affiliations:** Department of Ophthalmology, Nagoya University Graduate School of Medicine, Nagoya, Japan; Massachusetts Eye & Ear Infirmary, Harvard Medical School, UNITED STATES

## Abstract

**Purpose:**

Vessel maturation is considered to proceed by pruning branches resulting in less branching vessels. This study investigated the vessel junction densities of type 1 and type 2 choroidal neovascularizations (CNVs) using optical coherence tomography angiography (OCTA).

**Methods:**

We collected consecutive data from treatment-naïve eyes diagnosed with typical age-related macular degeneration (AMD). The OCTA images with CNV were analyzed to calculate vessel areas, vessel lengths, and vessel junction densities.

**Results:**

Of 60 eyes in 60 patients, type 1 CNV diagnoses had been made in 40 eyes, and type 2 CNV in 20 eyes. We found no significant difference in vessel areas between type 1 CNV and type 2 CNV (type 1 CNV, 0.44 ± 0.37 mm^2^; type 2 CNV, 0.37 ± 0.48 mm^2^), and no significant difference in vessel lengths (type 1 CNV, 18.24 ± 15.96 mm; type 2 CNV, 16.13 ± 21.45 mm). However, the vessel junction density of type 1 CNV was significantly lower than that of type 2 CNV by 16.0% (P = 0.008).

**Conclusion:**

OCTA revealed that the vessel junction densities of type 1 CNVs were lower than those of type 2 CNVs, suggesting type 1 CNV vessels are more mature than type 2 CNV vessels.

## Introduction

Neovascular age-related macular degeneration (AMD) is a vision-threatening disease characterized by pathological macular neovascularization. AMD is categorized into subtypes according to the origin and location of neovascular vessels [1]. Type 1 choroidal neovascularization (CNV) refers to vessels beneath the retinal pigment epithelium (RPE). Type 2 CNV refers to vessels expanding into the subretinal space between the neurosensory retina and the RPE. The gold standard treatment for typical AMD is the intravitreal injection consisting of anti-vascular endothelial growth factor (VEGF) agents, such as aflibercept and ranibizumab, because the treatment resolves CNV-related exudates and improves or maintains visual acuity in most patients with AMD [2–4]. However, it is known that complete regression of CNV is difficult to achieve using anti-VEGF monotherapy in most cases [5].

Optical coherence tomography angiography (OCTA) is now available in clinical settings. OCTA enables us to visualize retinal, and to some extent, choroidal vessels and provides three-dimensional angiographic images with high resolution by detecting erythrocyte movement in repeating A-scans at the same position [6–8]. OCTA improves the study of the CNV microvascular morphology because the alternative techniques, fluorescein angiography (FA) and indocyanine green angiography (ICGA), provide lower resolution and suffer from dye leakage from lesions [9, 10]. Studies have described OCTA images of type 1 CNV as having “sea fan,” “medusa,” or “tangled” patterns [11, 12]; while type 2 CNV has been reported as a branching networks of dense smaller caliber vessels radiating from the main trunk with a sharp demarcation [13, 14]. These studies have attempted to characterize the CNV microvascular morphology by appearance patterns, but qualitative and quantitative CNV analyses such as junction density and fractal dimension analyses are needed to identify biomarkers in order to understand the microvascular morphology and to predict activity and prognosis of CNV [15–17]. In general, angiogenesis proceeds by sprouting endothelial cells in a mechanism controlled by VEGF as the master switch of angiogenesis induction [18]. At the same time, vessel remodeling and maturation proceed by pruning excessive immature VEGF-dependent vessels and inducing hierarchical vessels with fewer branches [19, 20]. The surviving mature vessels are VEGF-independent, with little branching and large vessel diameter [21]. We recently studied CNV microvascular response to anti-VEGF therapy, focusing on the vessel junction density (i.e., the density of vessel branch points), and we found that mature VEGF-independent vessels were present even in treatment-naïve CNV [17]. Thus, we hypothesized that there is a difference in vessel maturation between treatment-naïve type 1 and type 2 CNV. In this study, we performed quantitative and qualitative OCTA analyses on the vessel junction densities of eyes with treatment-naïve typical AMD, and assessed the microvascular characteristics of type 1 and type 2 CNV.

## Materials and methods

This was a retrospective observational case series study. Data from consecutive patients with treatment-naïve eyes who were newly diagnosed with typical AMD, but had neither polypoidal choroidal vasculopathy nor type 3 neovascularization, at Nagoya University Hospital from March 2016 to September 2018 were collected. We followed the tenets of the Declaration of Helsinki, received approval from the Institutional Review Board of the Nagoya University Graduate School of Medicine (2018–0254), and appropriately registered the study with the University Hospital Medical Information Network (UMIN000033408). The Institutional Review Board granted a waiver of informed consent for the present study because of its retrospective nature. We also published the study outline on the Nagoya University website to give patients the opportunity to decline participation in the present study. All the patient data were anonymized prior to analysis. For showing representative cases in the present study, the patients for these cases have given written informed consent (as outlined in PLOS consent form) to publish the case details.

Patients involved in the study underwent comprehensive ophthalmic examinations, including best-corrected visual acuity (BCVA), fundus photography, OCT (Spectralis, Heidelberg Engineering, Heidelberg, Germany), FA and ICGA (Spectralis, Heidelberg Engineering), and OCTA (AngioPlex; CIRRUS HD-OCT model 5000, Carl Zeiss Meditec, Jena, Germany). Clinical diagnoses were made by fundus photography, OCT, and FA/ICGA findings. We classified CNVs into type 1 and type 2 according to published disease definitions [1]. CNVs containing both type 1 and type 2 lesions were classified as type 2 for the purposes of this study. Data from patients treated with photodynamic therapy or intravitreal injection of anti-VEGF drugs, as well as that from patients with a history of vitrectomy, aphakia, uveitis in either eye, or non- or proliferative diabetic retinopathy were excluded from the study.

**Fig 1:**
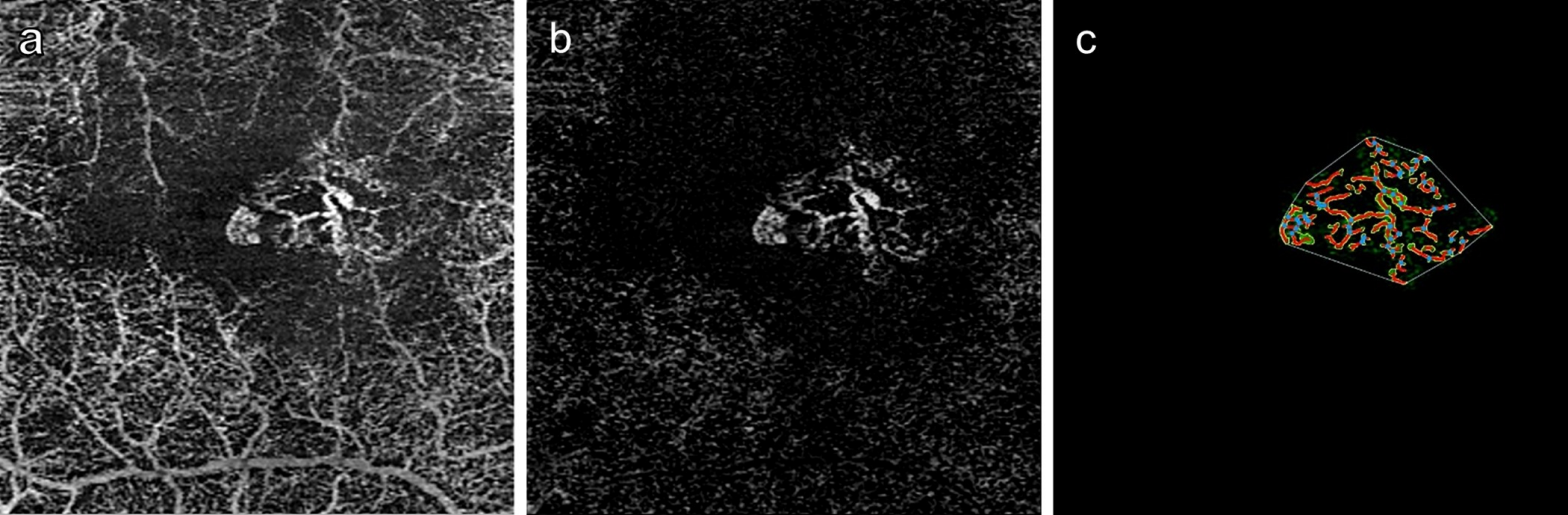
Quantitative analyses of choroidal neovascularization. (a) The CNV-containing slab was generated with the segmentation from the lower edge of the outer nuclear layer to either the retinal pigment epithelium or the line of Bruch’s membrane. (b) The CNV image free of projection artifacts was generated by subtraction of the retinal vessel image. (c) An open-source software (AngioTool) measured the vessel area surrounded by green lines, the vessel length indicated by red lines, and junction number with blue points.

We performed CNV evaluations as described in detail previously, with slight modifications [17]. In brief, we used macular 3 × 3-mm cubes scan patterns, including 245 A-scans per 3 mm, to assess the presence of CNV by OCTA. The patterns (. We manually chose the boundaries of CNV-containing slabs (CNV slab) from the lower edge of the outer nuclear layer to either the RPE or the line of Bruch’s membrane where the RPE line was disrupted or elevated, in order to generate custom en face images for the CNV analyses (Fig 1A). The slabs containing retinal vessels (retinal vessel slabs) were also generated by choosing the boundaries from the inner limiting membrane to the lower edge of the outer nuclear layer. Projection artifact-free CNV images were then generated by subtracting the retinal vessel slab images from the CNV slab images, using the GNU Image Manipulation Program (GIMP 2.8.14) (Fig 1B). An independent evaluator, masked from structural OCT and FA/ICGA images, processed the OCTA images and analyzed them using open-source software AngioTool (version 0.6a) [22]. In each case, the vessel areas and length as well as the number of vessel junctions were calculated (Fig 1C). Then, using these data, we calculated the vessel junction density per unit vessel length (total number of vessel junctions/total vessel lengths).

### Statistical analyses

All statistical analyses were performed with the SPSS, Version 24 (IBM, Armonk, NY, USA). We used Shapiro-Wilk tests to determine whether the data were normally distributed. The unpaired student’s t-test was used for the normally distributed data while the Mann-Whitney U test was applied to non-normally distributed data. We used χ^2^ tests for comparisons between the two groups, and the Spearman's correlation test to assess associations between factors. Data are presented by the mean ± standard deviation, and we considered all *P* < 0.05 as statistically significant.

## Results

We obtained data from 77 treatment-naïve eyes of 77 patients with typical AMD; of these, data from 17 eyes were excluded due to the low image quality of their OCTAs (caused by massive hemorrhage and exudates, or excess motion artifact). We ended up including data from a total of 60 eyes from 60 patients (41 men and 19 women). The mean age of the patients was 72.11 ± 8.85 years (range, 48–92 years), and the mean BCVA at the time of diagnosis was 0.33 ± 0.34 logMAR (Snellen visual acuity range, 20/500–20/20). According to the OCT and FA/ICGA images, 40 eyes were categorized into type 1 CNV and 20 eyes were categorized into type 2 CNV (6 of type 2 had both type 1 and type 2 CNVs at the lesions). The baseline characteristics of both patients with type 1 and with type 2 CNV are shown in Table 1. We found no significant differences in terms of age, gender, affected eye, BCVA, and symptom duration between the type 1 CNV and the type 2 CNV groups, although the age of patients in the type 2 CNV group tended to be younger (P = 0.057) and their symptom durations tended to be shorter (P = 0.075). We exclude 9 eyes (8 of type 1 CNV and 1 of type 2 CNV) from the analysis of symptom duration because their symptom duration was unclear. We found 4 eyes (all CNV type 1) that did not need anti-VEGF therapy, because their lesions lacked exudative changes and were defined as quiescent lesions.

**Table 1:** Patients’ demographics and characteristics.

Variables	Type 1 (n = 40)	Type 2 (n = 20)	*P* Value
Age (years)	73.65 ± 7.85 (58–92)	69.05 ± 10.08 (48–90)	0.057^a^
Sex (male/female)	28/12	13/7	0.695^b^
Affected eye (OS/OD)	20/20	8/12	0.464^b^
Symptom duration (days)	149.97 ± 209.93 (7–1008)	182.68 ± 421.36 (2–336)	0.075^c^
BCVA	0.30 ± 0.37 (20/500–20/20)	0.39 ± 0.29 (20/200–20/20)	0.080^c^
Exudative changes (+/-)	36/4	20/0	0.187^b^

OD, right eye; OS, left eye; BCVA, best corrected visual acuity. Data are presented as mean ± standard deviation unless otherwise indicated.

^a^Student t-test.

^b^χ^2^ test.

^c^Mann-Whitney U test.

**Fig 2:**
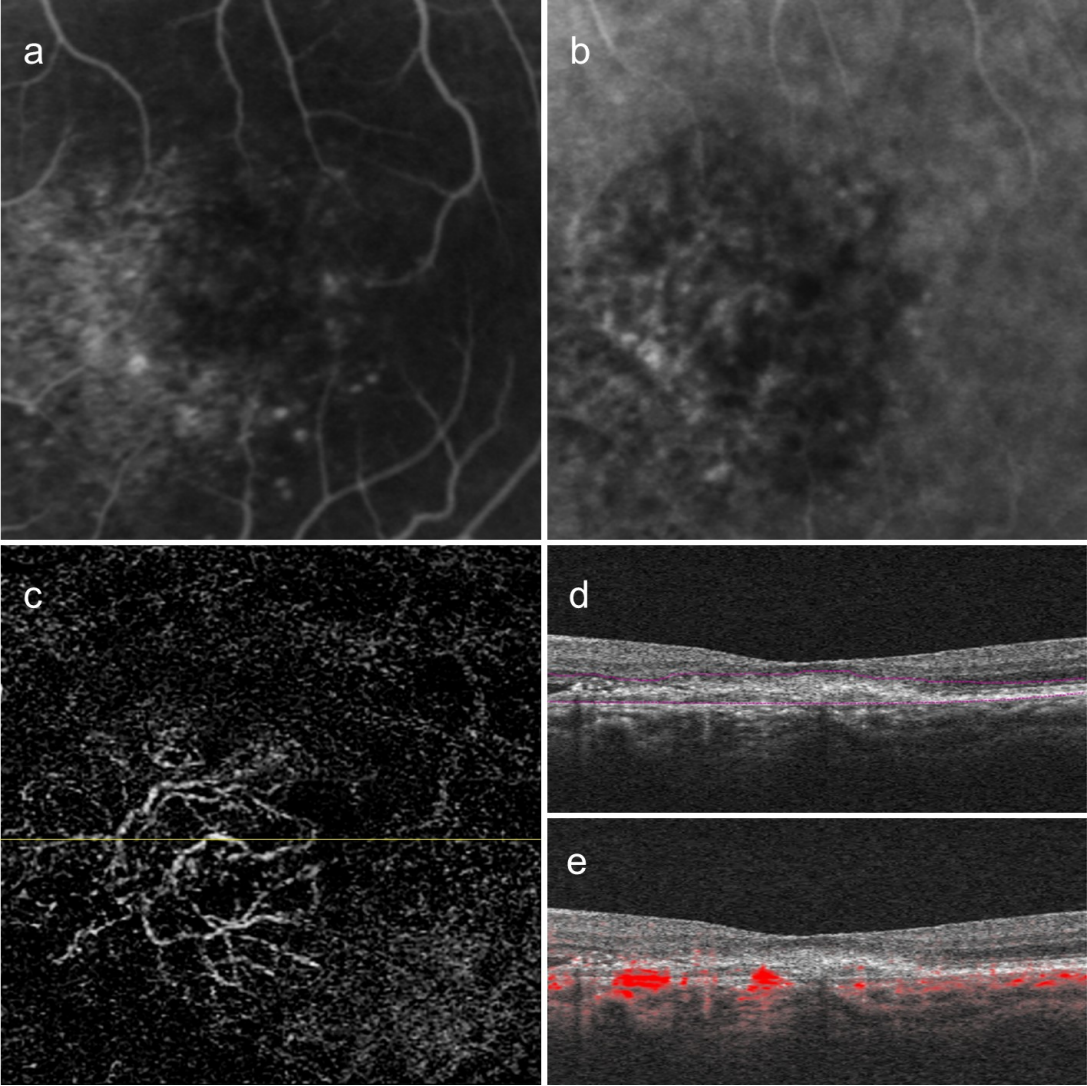
A case of Type 1 choroidal neovascularization. Left eye images of a 74-year-old woman with a visual acuity of 20/32. (a) Mid-phase fluorescein angiography showing leakage from an undetermined source. (b) Indocyanine green angiography showing a neovascular membrane. (c) 3 × 3-mm en face optical coherence tomography angiography (OCTA) images after removal of projection artifacts. The vessel branches radiate in all directions from the center of the lesion (medusa pattern). Note that the central vessels show trunk-like vessels. (d and e) Corresponding B-scan images showing segmentation lines (d) and flow signals overlaid on the B-scan image (e).

**Fig 3:**
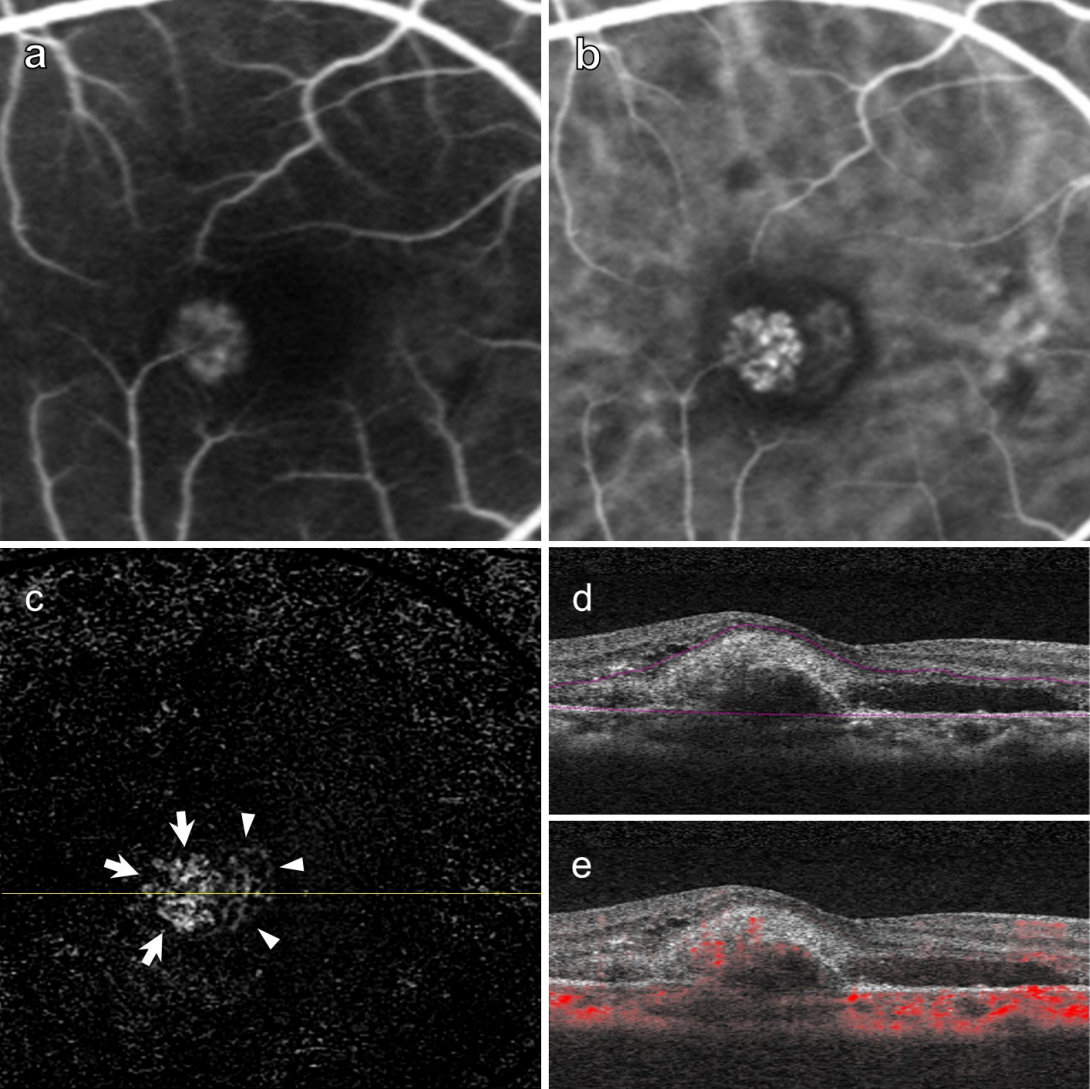
A case of Type 2 choroidal neovascularization (CNV). Right eye images of a 66-year-old woman with a visual acuity of 20/32. (a) Early-phase fluorescein angiography showing neovascular vessels that indicates a predominantly classic CNV. (b) Indocyanine green angiography showing the entire CNV. (c) 3 × 3-mm en face optical coherence tomography angiography (OCTA) images after removal of projection artifacts showing the dense small vessels. Note that the area corresponding with type 2 CNV (arrows) seems more vessel-dense compared to the area corresponding with type 1 CNV (arrow heads). (d) Corresponding B-scan images showing the segmentation lines. (e) Corresponding B-scan images with flow signals. Note the abnormal signals above and beneath the RPE, indicating that the lesion contains both type 1 and type 2 CNVs.

**Fig 4:**
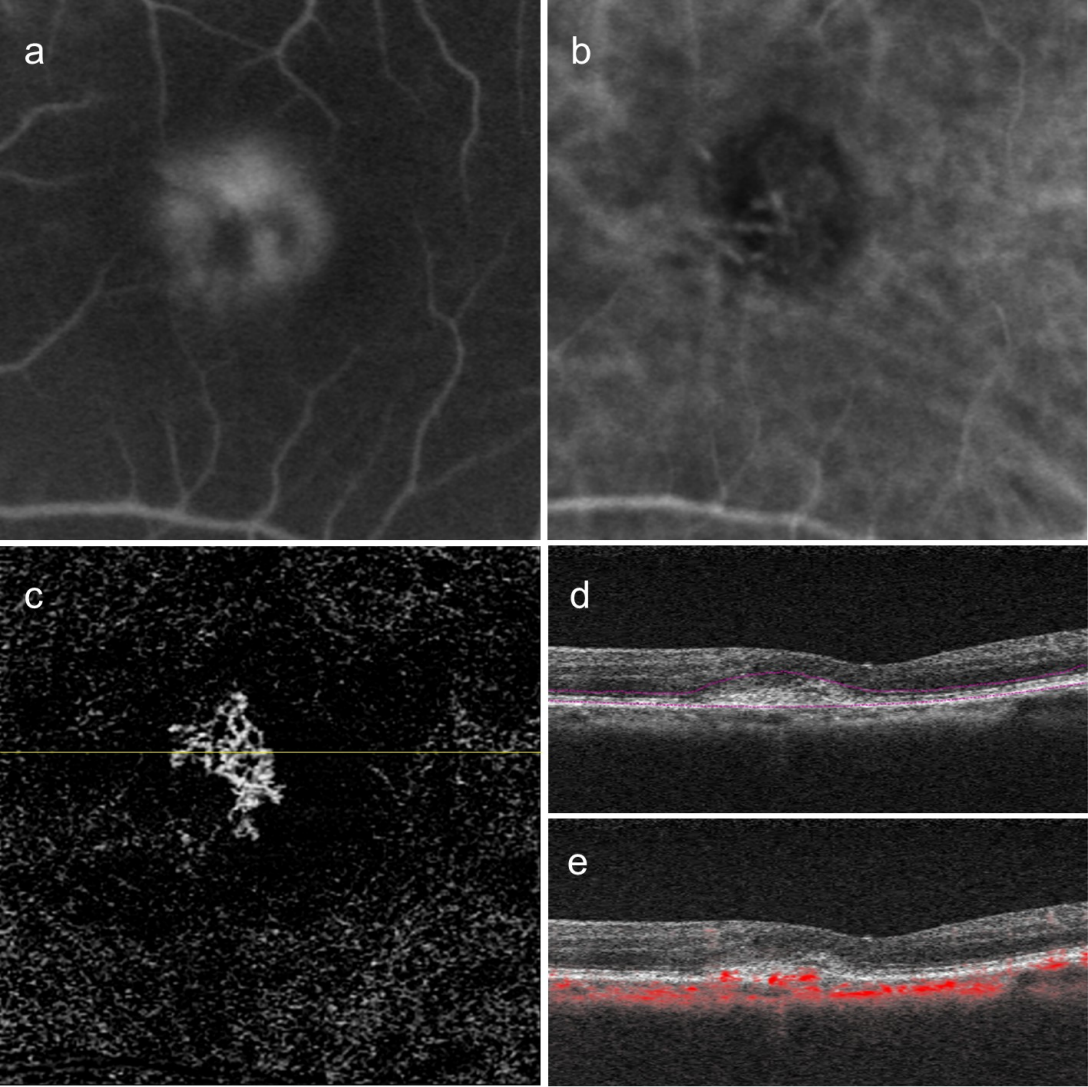
A case of Type 2 choroidal neovascularization (CNV). Left eye images of a 70-year-old female patient with a visual acuity of 20/25. (a) Mid-phase fluorescein angiography showing intense leakage and indicating a classic CNV type. (b) Indocyanine green angiography showing the neovascular vessels. (c) 3 × 3-mm en face optical coherence tomography angiography (OCTA) images after removal of projection artifacts showing the dense small vessels. (d and e) Corresponding B-scan images showing the segmentation lines (d) and flow signals overlaid on the B-scan image (e).

We found 25 of 40 type 1 CNV (62.5%) eyes had prominent trunk vessels at the center of the lesions. Representative cases of type 1 CNV eyes showed vessel branches radiating in all directions (medusa pattern) (Fig 2). The trunk vessels seemed to be located at the center of the lesion, but did not display appreciably large diameters. On the other hand, type 2 CNV eyes were likely to have dense, small branching vessels (Figs 3 and 4). A representative case of type 2 CNV in Fig 3 shows a lesion containing both type 1 and type 2 CNVs. The portion with type 2 CNV showed denser branching vessels than the adjacent portion with type 1 CNV. We did not identify a trunk vessel in this case. Fig 4 shows a representative case with only type 2 CNV lesions with dense small vessels. Of 20 eyes with type 2 CNV, we found prominent trunk vessels in 7 eyes (35%).

**Fig 5:**
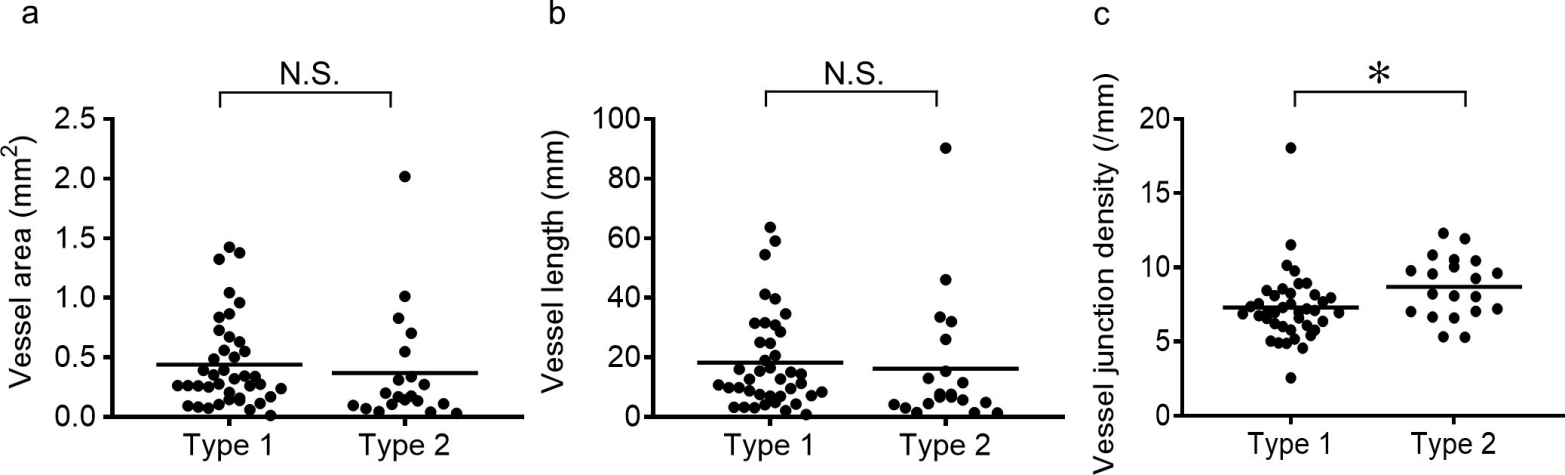
Quantitative and qualitative choroidal neovascularization (CNV) analyses. Vessel area (a), vessel length (b), and vessel junction density per length (c) of type 1 and type 2 CNVs. *Statistically significant difference (P < 0.05). N.S., not significant.

We performed qualitative and quantitative analyses of type 1 and type 2 CNVs as shown in Figs 2, 3, and 4. Vessel areas and lengths, and vessel junction densities per length were analyzed using the AngioTool software (Table 2, Fig 5). The mean vessel area of type 1 and type 2 were 0.44 ± 0.37 mm^2^ and 0.37 ± 0.48 mm^2^, respectively. The mean vessel lengths of type 1 and type 2 CNVs were 18.24 ± 15.96 mm and 16.13 ± 21.45 mm, respectively. We found no significant differences in the mean vessel area and the mean vessel length between type 1 and type 2 CNV groups (P = 0.11 and P = 0.17, respectively). One of the type 1 CNVs showed an extremely high vessel junction density (18.1/mm) with an extremely small lesion size (0.03 mm^2^) compared to those of the study population. The mean vessel junction density of type 2 CNVs (8.69 ± 2.05/mm) was significantly higher than that of type 1 CNVs (7.30 ± 2.40/mm, P = 0.008).

**Table 2:** Vessel area, vessel length, and vessel junction density on optical coherence tomography angiography in eyes with Type 1 and Type 2 choroidal neovascularization.

Variables	Type 1 (n = 40)	Type 2 (n = 20)	*P* Value
Vessel area (mm^2^)	0.44 ± 0.37 (0.01–1.43)	0.37 ± 0.48 (0.03–2.02)	0.106
Vessel length (mm)	18.24 ± 15.96 (0.78–63.65)	16.13 ± 21.45 (1.32–90.28)	0.168
Vessel junction density (/mm)	7.30 ± 2.40 (2.56–18.06)	8.69 ± 2.05 (5.30–12.31)	0.008*

Data are presented as mean ± standard deviation unless otherwise indicated.

Mann-Whitney U test was used when comparing categorical variable between Type 1 and Type 2.

*P < 0.05.

## Discussion

OCTA can be used to evaluate the microvascular CNV structures that are difficult to visualize by FA/ICGA due to the obscure images obtained from dye leakage and the low image resolution [8]. Being able to evaluate microvascular structures mean we may possibly be able to predict vessel quality (anti-VEGF therapy resistance or vascular maturation). In this study, we demonstrated that the vessel junction densities of treatment-naïve type 1 CNVs were lower than those of treatment-naïve type 2 CNVs. This suggests that type 1 CNVs may contain more mature vessels than type 2 CNVs, even when they are found in treatment-naïve eyes.

Spaide showed that type 1 CNVs treated with repeated anti-VEGF therapies showed large-caliber trunk vessels, and branching and loop vessels; and he explained that anti-VEGF medication regresses newly growing vascular sprouts, causing remaining vessels to increase their blood flow and the vessel diameter [15]. Kuehlewein et al reported similar findings and described the type 1 CNV patterns as medusa- or sea fan-like [12]. In our study, we found prominent trunk vessels in 62.5% of type 1 CNVs, although the average vessel diameter was smaller than those in the literature [12]. In particular, for us it was difficult to find trunk vessels in type 2 CNVs (we found them in only 35% of type 2 CNVs). This difference may be due to our focus on treatment-naïve eyes in which the remodeling of the vessels may not have been completed as it probably was in eyes treated with multiple anti-VEGF injections.

In a comparison of treatment-naïve and treated eyes, Sulzbacher et al reported that 12 of 14 treatment-naïve eyes with CNV showed dense vessel-net patterns, and 74 eyes treated repeatedly with anti-VEGF treatment showed loose net CNV patterns with larger vessel diameters and a low branching index associated with long disease duration [23]. In our study, we had hypothesized that the more mature vessels had been present for a longer time. Thus, we analyzed the symptom duration between type 1 CNV and type 2 CNV at the time of diagnosis. Our results showed that the symptom duration of patients with type 1 CNV tended to be longer than that of patients with type 2 CNV, but we found no statistical significance. It is difficult to speculate on the precise CNV duration from the symptom duration, because some CNVs develop asymptomatically. It has been reported that asymptomatic type 1 CNV was detected using OCTA[24] [25]. Although immature CNV is leaky and active, small exudative changes beneath RPE may not damage neurosensory retina and not cause any symptom. Whereas, type 2 CNV cause intense vision worsening, because it extends into the subretinal space, damaging the neurosensory retina directly. Thus, eyes with treatment-naïve type 2 CNV may be diagnosed earlier than those with treatment-naïve type 1 CNV, which would be the reason why treatment-naïve type 2 CNVs contain more immature vessels than treatment-naïve type 1 CNVs.

Newly formed vessels are not covered with pericytes and smooth muscle cells, and are relatively dependent on VEGF signals [26]. Once covered with pericytes and smooth muscle cells, which are called mature vessels, they become VEGF-independent and refractory to anti-VEGF therapy. We recently reported that, even in treatment-naïve eyes, some CNV lesion parts are resistant to aflibercept injection, suggesting the presence of VEGF-independent mature vessels even before the first treatment [17]. In addition, it is known that type 1 CNV tends to be resistant against anti-VEGF therapy, which is in part surmised to be due to the fact that RPE covering type 1 CNV may block penetration of anti-VEGF drugs [27]. In this study, we revealed that type 1 CNVs have more mature vessels than type 2 CNVs. Thus, vessel maturation may be a reason for the anti-VEGF resistance of type 1 CNVs.

We aware of this study’s limitations that include the use of a novel technology. OCTA provides angiographic images by detecting motion contrast from flowing blood. Eye movements, the blocking effect by exudates, and projections may cause artifacts [28]. Some of the patients in this study had difficulty maintaining the eye position because of the central vision disturbance, even though we used the fundus tracking system in the OCTA application. Some of the lesions were covered with massive exudates and hemorrhage, which blocked the penetration of OCT signals. Thus, we excluded about 20% of the eyes from the analyses, which may have created a selection bias. In addition, the removal of projection artifacts may also cause loss of signal from CNVs. More advanced technologies are required to eliminate such artifacts. Another limitation of the present study is that OCTA cannot detect extremely slow blood flow due to the scanning speed and its algorism. Thus, the vessels analyzed in the present study may be underestimated. Vessels with extremely slow blood flow may be about to close the lumens of the vessels, followed by pruning of the vessels, which is considered as a part of vessel maturation. OCTA is now developing rapidly, with better resolution and better algorithms. Future versions of OCTA will reveal more detailed vessels of extremely slow blood.

This study provides the largest OCTA analysis of treatment-naïve CNV secondary AMD to date, and compares both qualitative and quantitative analysis of type 1 and type 2 CNVs for the first time. Our findings suggest that treatment-naïve type 1 CNVs contain more mature vessels than treatment-naïve type 2 CNVs, which may explain the different responses to anti-VEGF therapy. This evidence is an important clue not only to understand recalcitrant CNV, but also for finding new treatment strategies for long-term management of CNV.

## Supporting information

S1 Table Original data including types of CNV, symptom duration, vessel area, vessel length, and vessel junction density. (XLSX)
